# Geometrical Nonlinearity for a Timoshenko Beam with Flexoelectricity

**DOI:** 10.3390/nano11113123

**Published:** 2021-11-19

**Authors:** Miroslav Repka, Jan Sladek, Vladimir Sladek

**Affiliations:** Institute of Construction and Architecture, Slovak Academy of Sciences, Dubravska Cesta 9, 84503 Bratislava, Slovakia; jan.sladek@savba.sk (J.S.); vladimir.sladek@savba.sk (V.S.)

**Keywords:** von kármán large deformations, flexoelectricity, cantilever beam, timoshenko model, nonlinear system

## Abstract

The Timoshenko beam model is applied to the analysis of the flexoelectric effect for a cantilever beam under large deformations. The geometric nonlinearity with von Kármán strains is considered. The nonlinear system of ordinary differential equations (ODE) for beam deflection and rotation are derived. Moreover, this nonlinear system is linearized for each load increment, where it is solved iteratively. For the vanishing flexoelectric coefficient, the governing equations lead to the classical Timoshenko beam model. Furthermore, the influence of the flexoelectricity coefficient and the microstructural length-scale parameter on the beam deflection and the induced electric intensity is investigated.

## 1. Introduction

The size effect on micro/nano structural elements is observed due to the comparable sizes of these elements and the material microstructural length-scale. Classical continuum models include only the first-order gradients of primary fields and do not reflect the material microstructure. Therefore, these models are inapplicable of describing and explaining the phenomena (e.g., flexoelectricity), where the second-order gradients play a role. On the other hand, higher-grade continuum theories contain higher gradients of primary fields. In addition, the additional coefficients in constitutive relationships are determined by the material microstructure, which is usually accomplished through the microstructural length-scale parameter. Buckling of centrosymmetric anisotropic beams was analyzed in Reference [1] with the strain-gradient theory. Then, the geometric nonlinearity with von Kármán strains [2] are taken into account. Wang et al. [3] analyzed the nonlinear free vibration of electrically actuated nanobeams, where the surface energy, temperature change, geometrical nonlinear deformation, and intermolecular Casimir force are considered.

Thin beams are commonly used in nanoelectromechanical devices and for energy harvesting [4]. Traditionally, the piezoelectricity is utilized to convert mechanical energy to electrical energy or vice versa [5]. In piezoelectric materials, a uniform mechanical strain can induce an electric polarization. This conversion is observed only in non-centrosymmetric crystal structures. Numerous crystalline materials are not piezoelectric since their structure is centrosymmetric. However, a non-uniform strain or the presence of strain-gradients may potentially break the inversion symmetry and induce polarization, even in centrosymmetric crystals [6,7,8]. In the literature, this phenomenon is called the flexoelectric effect [9,10]. Flexoelectric energy harvesters are intensively studied in [11,12,13]. A rectangular beam is the most common model used for this purpose. The Euler–Bernoulli and Timoshenko beam theories are applied to analyze the beams. In the Euler–Bernoulli theory, there are vanishing shear strain deformations. The Timoshenko beam model based on the strain-gradient elasticity theory and the couple stress elasticity theory [14,15] has been developed only recently [16]. The microstructure-dependent Timoshenko beam model contains a material length-scale parameter and can capture the size effect. A flexoelectric Euler–Bernoulli model for energy harvesting is proposed in [17]. The first attempt to consider the geometric nonlinearity deformation in flexoelectricity has been developed by Wang and Wang [18] with a beam described by the Euler–Bernoulli theory. The authors used simplification, where the gradient of normal strain along the beam vanishes. This leads to the simplification of the nonlinear system of differential equations. An exact solution is proposed for the nonlinear forced vibration analysis of nanobeams made of functionally graded materials (FGMs) subjected to a thermal environment, including the effect of surface stress in [19]. Thai et al. [20] proposed an isogeometric approach for general problems of flexoelectricity in soft dielectric materials at finite deformations, accounting for Maxwell stresses. A nonlinear vibration of flexoelectric nanobeams with surface and thermal effects is investigated by the surface elasticity theory [21]. A similar nonlinear bending problem with Kármán nonlinear deformations for the coupled piezomagnetic–flexomagnetic nanosized Euler–Bernoulli beam is analyzed in [22]. Recently, they extended an early paper to the geometrically nonlinear vibration of the piezo-flexomagnetic nanotube [23]. Free vibrations of a visco-piezo-flexoelectric nanobeam are given in [24]. Sahmani and Aghdam [25] applied the nonlocal strain-gradient beam model with the third-order distribution of shear deformation to explore the nonlinear vibration of axially-loaded multilayer functionally graded nanobeams in both the pre-buckling and post-buckling domains. A similar nanoplate problem for the electro-mechanical shear buckling analysis by the modified couple stress theory with various boundary conditions is given in [26].

In the present paper, von Kármán large deformations in the direct flexoelectricity are considered for a cantilever beam without simplification. The Timoshenko model with geometrical nonlinearity is applied to derive the nonlinear system of ordinary differential equations (ODEs) for the beam deflection and rotation. The mechanical load in the nonlinear system is considered incrementally. In each increment, the system of ODEs should be linearized, where nonlinear terms are considered iteratively.

## 2. A Linear Theory of Direct Flexoelectricity

The electric enthalpy density for piezoelectric solids with the direct flexoelectricity can be written as [8,27]:

(1) $$H=\frac{1}{2}c_{ijkl}\varepsilon_{ij}\varepsilon_{kl}-\frac{1}{2}a_{ij}E_{i}E_{j}-e_{kji}\varepsilon_{ij}E_{k}+\frac{1}{2}g_{jklmni}\eta_{jkl}\eta_{mni}-f_{ijkl}E_{i}\eta_{jkl},$$

where the symbols *a* and *c* are used for the second-order permittivity and the fourth-order elastic constant tensors, respectively. The piezoelectric coefficient is denoted by symbol *e*, and symbol *f* is the direct flexoelectric coefficient. The tensor *g* is used for higher-order elastic coefficients representing the strain-gradient elasticity.

The strain tensor $\varepsilon_{ij}$ and the electric field vector $E_{j}$ are defined as [28]:

(2) $$\;\varepsilon_{ij}=\frac{1}{2}\left(u_{i,j}+u_{j,i}\right)\;,\;E_{j}=-\phi_{,j}\;,$$

where $u_{i}$ and ϕ are the displacements and electric potential, respectively.

The strain-gradient tensor $\eta_{ijk}$ is given by:

(3) $$\;\eta_{ijk}=\varepsilon_{ij,k}=\frac{1}{2}\left(u_{i,jk}+u_{j,ik}\right)\;,$$

Under the infinitesimal deformations, the constitutive equations can be obtained from the electric enthalpy density expression (1) [27,29]:

(4) $$\sigma_{ij}=\frac{\partial H}{\partial \varepsilon_{ij}}=c_{ijkl}\varepsilon_{kl}-e_{kij}E_{k},\;\tau_{jkl}=\frac{\partial H}{\partial \eta_{jkl}}=-f_{ijkl}E_{i}+g_{jklmni}\eta_{nmi},\;D_{i}=-\frac{\partial H}{\partial E_{i}}=a_{ij}E_{j}+e_{ijk}\varepsilon_{jk}+f_{ijkl}\eta_{jkl}$$

where $\sigma_{ij}$, $D_{k}$, and $\tau_{jkl}$ are the stress tensor, electric displacements, and higher-order stress tensor, respectively.

The size scale of higher-order elastic parameters $g_{jklmni}$ is expressed by a proportionality of the conventional elastic stiffness coefficients $c_{klmn}$ and the microstructural length-scale parameter *l* [1,30]:

(5) $$g_{jklmni}=l^{2}c_{jkmn}\delta_{li},$$

with $\delta_{li}$ as the Kronecker delta.

Deng et al. [31] considered two independent components $f_{1}$ and $f_{2}$ for the direct flexoelectric coefficient $f_{ijkl}$, $f_{ijkl}=f_{1}\delta_{jk}\delta_{il}+f_{2}\left(\delta_{ij}\delta_{kl}+\delta_{ik}\delta_{jl}\right)$. Then, the electric enthalpy density has the following form:

(6) $$H=\frac{1}{2}c_{ijkl}\varepsilon_{ij}\varepsilon_{kl}-\frac{1}{2}a_{ij}E_{i}E_{j}-e_{kji}\varepsilon_{ij}E_{k}+\frac{l^{2}}{2}c_{jkmn}\eta_{jkl}\eta_{mnl}-f_{1}E_{i}\eta_{kki}-f_{2}E_{i}\left(\eta_{ikk}+\eta_{jij}\right),\;$$

If the poling direction is along the $x_{3}$-axis in the piezoelectric material, the electric enthalpy has the following form:

(7) $$H=\frac{1}{2}c_{ijkl}\varepsilon_{ij}\varepsilon_{kl}-\frac{1}{2}a_{ij}E_{i}E_{j}-e_{31}\varepsilon_{11}E_{3}-e_{33}\varepsilon_{33}E_{3}-e_{15}\left(\varepsilon_{13}+\varepsilon_{31}\right)E_{1}+\frac{l^{2}}{2}c_{jkmn}\eta_{jkl}\eta_{mnl}\;-f_{1}E_{i}\eta_{kki}-f_{2}E_{i}\left(\eta_{ikk}+\eta_{jij}\right),$$

The constitutive Equation (4) for orthotropic materials,

$$c_{ijkl}=\delta_{i1}\delta_{j1}\left(c_{11}\delta_{k1}\delta_{l1}+c_{13}\delta_{k3}\delta_{l3}\right)+\delta_{i3}\delta_{j3}\left(c_{13}\delta_{k1}\delta_{l1}+c_{33}\delta_{k3}\delta_{l3}\right)+c_{44}\left(\delta_{i1}\delta_{j3}+\delta_{i3}\delta_{j1}\right)\left(\delta_{k1}\delta_{l3}+\delta_{k3}\delta_{l1}\right),\;a_{ij}=a_{1}\delta_{i1}\delta_{j1}+a_{3}\delta_{i3}\delta_{j3}$$

can be rewritten into a matrix form as in [32]:

(8) $$\left[\begin{array}{c} \sigma_{11}\\ \sigma_{33}\\ \sigma_{13} \end{array}\right]=\left[\begin{array}{ccc} c_{11} & c_{13} & 0\\ c_{13} & c_{33} & 0\\ 0 & 0 & c_{44} \end{array}\right]\left[\begin{array}{c} \varepsilon_{11}\\ \varepsilon_{33}\\ 2\varepsilon_{13} \end{array}\right]-\left[\begin{array}{c} \begin{array}{cc} 0 & e_{31} \end{array}\\ \begin{array}{cc} 0 & e_{33} \end{array}\\ \begin{array}{cc} e_{15} & 0 \end{array} \end{array}\right]\left[\begin{array}{c} E_{1}\\ E_{3} \end{array}\right]=C\left[\begin{array}{c} \varepsilon_{11}\\ \varepsilon_{33}\\ 2\varepsilon_{13} \end{array}\right]-\Lambda \left[\begin{array}{c} E_{1}\\ E_{3} \end{array}\right],$$

(9) $$\left[\begin{array}{c} D_{1}\\ D_{3} \end{array}\right]=\left[\begin{array}{ccc} \begin{array}{c} 0\\ e_{31} \end{array} & \begin{array}{c} 0\\ e_{33} \end{array} & \begin{array}{c} e_{15}\\ 0 \end{array} \end{array}\right]\left[\begin{array}{c} \varepsilon_{11}\\ \varepsilon_{33}\\ 2\varepsilon_{13} \end{array}\right]+\left[\begin{array}{cc} a_{1} & 0\\ 0 & a_{3} \end{array}\right]\left[\begin{array}{c} E_{1}\\ E_{3} \end{array}\right]+\left[\begin{array}{cccccc} f_{1}+2f_{2} & f_{1} & 0 & 0 & 0 & f_{2}\\ 0 & 0 & f_{2} & f_{1} & f_{1}+2f_{2} & 0 \end{array}\right]\left[\begin{array}{c} \eta_{111}\\ \eta_{331}\\ 2\eta_{131}\\ \eta_{113}\\ \eta_{333}\\ 2\eta_{133} \end{array}\right]\;=\Lambda^{T}\left[\begin{array}{c} \varepsilon_{11}\\ \varepsilon_{33}\\ 2\varepsilon_{13} \end{array}\right]+\Pi \left[\begin{array}{c} E_{1}\\ E_{3} \end{array}\right]+F\left[\begin{array}{c} \eta_{111}\\ \eta_{331}\\ 2\eta_{131}\\ \eta_{113}\\ \eta_{333}\\ 2\eta_{133} \end{array}\right]$$

(10) $$\left[\begin{array}{c} \tau_{111}\\ \tau_{331}\\ \tau_{131}\\ \tau_{113}\\ \tau_{333}\\ \tau_{133} \end{array}\right]=-\left[\begin{array}{cc} f_{1}+2f_{2} & 0\\ f_{1} & 0\\ 0 & f_{2}\\ 0 & f_{1}\\ 0 & f_{1}+2f_{2}\\ f_{2} & 0 \end{array}\right]\left[\begin{array}{c} E_{1}\\ E_{3} \end{array}\right]+l^{2}\left[\begin{array}{cccccc} c_{11} & c_{13} & 0 & 0 & 0 & 0\\ c_{13} & c_{33} & 0 & 0 & 0 & 0\\ 0 & 0 & c_{44} & 0 & 0 & 0\\ 0 & 0 & 0 & c_{11} & c_{13} & 0\\ 0 & 0 & 0 & c_{13} & c_{33} & 0\\ 0 & 0 & 0 & 0 & 0 & c_{44} \end{array}\right]\left[\begin{array}{c} \eta_{111}\\ \eta_{331}\\ 2\eta_{131}\\ \eta_{113}\\ \eta_{333}\\ 2\eta_{133} \end{array}\right]=-F^{T}\left[\begin{array}{c} E_{1}\\ E_{3} \end{array}\right]+l^{2}G\left[\begin{array}{c} \eta_{111}\\ \eta_{331}\\ 2\eta_{131}\\ \eta_{113}\\ \eta_{333}\\ 2\eta_{133} \end{array}\right],\;$$

The governing equations for the piezoelectric solid with direct flexoelectric effects are given in [33]:

(11) $$\sigma_{ij,j}(x)-\tau_{ijk,jk}(x)=0,\;D_{i,i}(x)=0$$

Moreover, one can find the form of essential and natural boundary conditions (b.c.):

1. Essential b.c.:
  (12) $$u_{i}(x)={\bar{u}}_{i}(x)\;\mathrm{on}\;\Gamma_{u},\;\Gamma_{u}\subset \Gamma ,\;s_{i}(x)={\bar{s}}_{i}\;\mathrm{on}\;\Gamma_{s},\Gamma_{s}\subset \Gamma ,\;\phi (x)=\bar{\phi }(x)\;\mathrm{on}\;\Gamma_{\phi },\Gamma_{\phi }\subset \Gamma$$
2. Natural b.c.:
  (13) $$t_{i}(x)={\bar{t}}_{i}(x)\;\mathrm{on}\;\Gamma_{t},\;\Gamma_{t}\cup \Gamma_{u}=\Gamma ,\;\Gamma_{t}\cap \Gamma_{u}=\emptyset ,\;R_{i}(x)={\bar{R}}_{i}(x)\mathrm{on}\;\Gamma_{R},\;\Gamma_{R}\cup \Gamma_{s}=\Gamma ,\;\Gamma_{R}\cap \Gamma_{s}=\emptyset ,\;S(x)=\bar{S}(x)\;\mathrm{on}\;\Gamma_{S},\;\Gamma_{S}\cup \Gamma_{\phi }=\Gamma ,\;\Gamma_{S}\cap \Gamma_{\phi }=\emptyset$$
  where
  (14) $$s_{i}:=\frac{\partial u_{i}}{\partial n},\;R_{i}:=n_{k}n_{j}\tau_{ijk},$$
  the traction vector and the electric charge are defined as follows:
  (15) $$t_{i}:=n_{j}\left(\sigma_{ij}-\tau_{ijk,k}\right)-\frac{\partial \rho_{i}}{\partial \pi }+\sum_{c}\left\|\rho_{i}(x^{c})\right\|\delta (x-x^{c}),$$
  (16) $$S:=n_{k}D_{k},\;$$
  with $\rho_{i}:=n_{k}\pi_{j}\tau_{ijk}$, δ(x) is the Dirac delta function, and $\pi_{i}$ is the Cartesian component of the unit tangent vector on Γ.

The jump at a corner (**x***^c^*) on the oriented boundary contour Γ is defined as:

(17) $$\left\|\rho_{i}(x^{c})\right\|:=\rho_{i}(x^{c}-0)-\rho_{i}(x^{c}+0)$$

Regarding the electric boundary conditions, the prescription of the electric potential (as given by (12)) also includes the short-circuit case, when the electric potential is constant on the whole boundary (vanishing). On the other hand, in the case of open-circuit conditions, the electric potential is unknown and a certain value of the free electric charge on the boundary is prescribed. If the case of the short-circuit condition is applied to the beam problem considered in the next section, the electric field *E*_3_ is equal to zero. Therefore, the open-circuit conditions are applied.

## 3. Timoshenko Beam with Flexoelectric Effect and Nonlinear Strains

**Figure 1:**
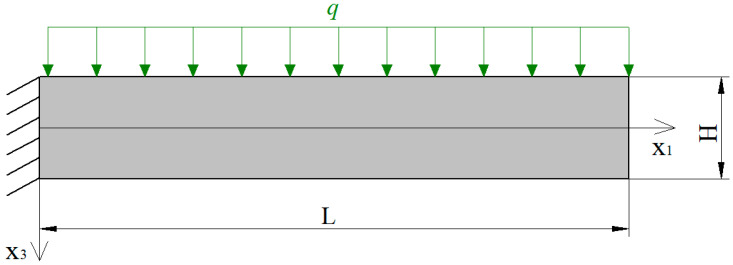
Cantilever beam under the transverse force load.

Next, we analyze a cantilever beam by taking into account the direct flexoelectric effect and large von Kármán strains (geometric nonlinear deformation). The $x_{1}$-axis is the neutral axis of the undeformed beam, and the $x_{3}$-axis is along the thickness direction. A uniform transverse load *q* is applied on the upper surface of the cantilever beam (Figure 1).

In the traditional Timoshenko beam theory with finite shear stresses, the displacement of the beam can be expressed as:

(18) $$u_{1}(x_{1},x_{3})=-x_{3}\phi (x_{1}),\;u_{2}(x_{1},x_{3})=0,\;u_{3}(x_{1},x_{3})=w(x_{1}),\;$$

where $w(x_{1})$ is the transverse displacement of the neutral axis and $\phi (x_{1})$ is the rotation of the cross-section. With the assumption of very small slopes in the beam after deformation but a possible finite transverse deflection, the von Kármán nonlinear strain of the beam [1,2] is:

(19) $$\varepsilon_{11}=-x_{3}\phi_{,1}+\frac{1}{2}{\left(w_{,1}\right)}^{2},\;\varepsilon_{13}=\varepsilon_{31}=\frac{1}{2}\left(-\phi +w_{,1}\right),\;\varepsilon_{33}=0$$

The only non-zero strain-gradients are given as:

(20) $$\eta_{111}=-x_{3}\phi_{,11}+w_{,1}w_{,11},\;\eta_{113}=-\phi_{,1},\;\eta_{131}=\eta_{311}=\frac{1}{2}\left(-\phi_{,1}+w_{,11}\right)$$

Wang and Wang [18] simplified their solution neglecting $\eta_{111}=\varepsilon_{11,1}$, as compared to $\eta_{113}$. All of the three components of strain gradients in (20) are considered in this work.

For the beam, we assume that only the $E_{3}$ electric intensity component is non-vanishing. In the case of open-circuit condition, the electric displacement on the surface is vanishing. Therefore, $D_{3}=0$ on $x_{3}=\pm H/2$, and from the Maxwell equation for the electric displacement $D_{3,3}=0$, we obtain $D_{3}=0$ inside the beam. Then, from the constitutive Equation (9), we directly obtain the expression for the electric intensity component $E_{3}$.

(21) $$E_{3}=-\frac{e_{31}}{a_{3}}\varepsilon_{11}-\frac{f_{1}}{a_{3}}\eta_{113}-\frac{2f_{2}}{a_{3}}\eta_{131}.$$

In the case of the short-circuit condition, the electric intensity component $E_{3}$ is equal to zero. Then, the stress components are dependent only on the strains. In this case, finite values of higher-order stress tensors are found in (4), only due to the non-vanishing higher-order elastic parameters $g_{jklmni}$. In addition, the governing Equation (11) for mechanical quantities (beam deflection) is dependent on the electrical fields induced by flexoelectricity. If $g_{jklmni}$ are vanishing, the governing equation for the beam deflection is similar to the classical elasticity without electro-mechanical coupling [34].

Next, the open-circuit conditions are considered. Substituting the electric intensity component $E_{3}$ (21) into the stress (8) and higher-order stress tensors (10), we obtain:

(22) $$\begin{array}{l} \sigma_{11}=\left(c_{11}+\frac{e_{31}e_{31}}{a_{3}}\right)\varepsilon_{11}+\frac{e_{31}f_{1}}{a_{3}}\eta_{113}+\frac{2e_{31}f_{2}}{a_{3}}\eta_{131}\\ \sigma_{33}=\left(c_{13}+\frac{e_{33}e_{31}}{a_{3}}\right)\varepsilon_{11}+\frac{e_{33}f_{1}}{a_{3}}\eta_{113}+\frac{2e_{33}f_{2}}{a_{3}}\eta_{131}\\ \sigma_{13}=2c_{44}\varepsilon_{13} \end{array}$$

$$\tau_{111}=l^{2}c_{11}\eta_{111},$$

(23) $$\begin{array}{l} \tau_{331}=l^{2}c_{13}\eta_{111}\\ \tau_{131}=2l^{2}c_{44}\eta_{131}+f_{2}\left(\frac{e_{31}}{a_{3}}\varepsilon_{11}+\frac{f_{1}}{a_{3}}\eta_{113}+\frac{2f_{2}}{a_{3}}\eta_{131}\right)\\ \tau_{113}=l^{2}c_{11}\eta_{113}+f_{1}\left(\frac{e_{31}}{a_{3}}\varepsilon_{11}+\frac{f_{1}}{a_{3}}\eta_{113}+\frac{2f_{2}}{a_{3}}\eta_{131}\right)\\ \tau_{333}=l^{2}c_{13}\eta_{113}+(f_{1}+2f_{2})\left(\frac{e_{31}}{a_{3}}\varepsilon_{11}+\frac{f_{1}}{a_{3}}\eta_{113}+\frac{2f_{2}}{a_{3}}\eta_{131}\right)\\ \tau_{133}=0 \end{array}$$

The variational principle for this special case with a vanishing electric displacement has the following form:

$$\delta \int_{V}\left(ℱ-W\right)dV=\int_{0}^{L}\int_{A}\left(\sigma_{11}\delta \varepsilon_{11}+2\sigma_{13}\delta \varepsilon_{13}+\tau_{111}\delta \eta_{111}+\tau_{113}\delta \eta_{113}+2\tau_{131}\delta \eta_{131}\right)dAdx_{1}-\int_{0}^{L}q(x)\delta wdx_{1}=0$$

thus with the use of strain (19) and strain-gradient (20) expressions, we obtain:

(24) $$\begin{array}{l} \int_{0}^{L}\int_{A}\{-x_{3}\tau_{111}\delta \phi_{,11}-\left(x_{3}\sigma_{11}+\tau_{113}+\tau_{131}\right)\delta \phi_{,1}-\sigma_{13}\delta \phi +\left(w_{,1}\tau_{111}+\tau_{131}\right)\delta w_{,11}\\ +\left(\sigma_{11}w_{,1}+\sigma_{13}+w_{,11}\tau_{111}\right)\delta w_{,1}\}dAdx_{1}-\int_{0}^{L}q(x)\delta wdx_{1}=0 \end{array}$$

where *W* is the work of the external transverse load *q*(*x*) and *L* is the beam length.

Defining the bending moment, shear and normal forces, as well as their higher-order counterparts,

(25) $$M=\int_{A}\sigma_{11}x_{3}dA,\;Q=\int_{A}\sigma_{13}dA,\;N=\int_{A}\sigma_{11}dA,\;M^{H}=\int_{A}\tau_{111}x_{3}dA,\;Q^{H}=\int_{A}\tau_{131}dA,\;N^{H}=\int_{A}\tau_{111}dA,\;P^{H}=\int_{A}\tau_{113}dA,$$

we obtain:

(26) $$\int_{0}^{L}\left[\begin{array}{l} M^{H}\delta \phi_{,11}+\left(M+P^{H}+Q^{H}\right)\delta \phi_{,1}+Q\delta \phi -\left(Q^{H}+N^{H}w_{,1}\right)\delta w_{,11}\\ -\left(Q+Nw_{,1}+N^{H}w_{,11}\right)\delta w_{,1}+q(x)\delta w \end{array}\right]dx_{1}=0$$

Applying the integration in parts to the above equation, we obtain:

(27) $$\begin{array}{c} {M^{H}\delta \phi_{,1}|}_{0}^{L}{+\left(M+P^{H}+Q^{H}-M_{,1}^{H}\right)\delta \phi |}_{0}^{L}-{\left(Q^{H}+N^{H}w_{,1}\right)\delta w_{,1}|}_{0}^{L}\\ -{\left[Q+Nw_{,1}+N^{H}w_{,11}-{\left(Q^{H}+N^{H}w_{,1}\right)}_{,1}\right]\delta w|}_{0}^{L}-\int_{0}^{L}\left[{\left(M+P^{H}+Q^{H}-M_{,1}^{H}\right)}_{,1}-Q\right]\delta \phi dx_{1}\\ +\int_{0}^{L}\left\{{\left[Q+Nw_{,1}+N^{H}w_{,11}-{\left(Q^{H}+N^{H}w_{,1}\right)}_{,1}\right]}_{,1}+q\right\}\delta wdx_{1}=0 \end{array}$$

Since variations δϕ and δw can be arbitrary in the interval [0,  L], we obtain two governing equations:

(28) $${\left(M+P^{H}+Q^{H}-M_{,1}^{H}\right)}_{,1}-Q=0,\;Q_{,1}+N_{,1}w_{,1}+Nw_{,11}-\left(Q_{,11}^{H}+N_{,11}^{H}w_{,1}+N_{,1}^{H}w_{,11}\right)=-q$$

Furthermore, the possible boundary conditions are obtained from Equation (27).

(i) Total shear force or the beam deflection vanishes at the end of the beam:
  $$Q+Nw_{,1}+N^{H}w_{,11}-{\left(Q^{H}+N^{H}w_{,1}\right)}_{,1}=0\;\;\;\;\;\;\mathrm{or}\;\;\;\;\;\;\;w=0\;\;\;\;\mathrm{at}\;\;\;x_{1}\in \left\{0,\;L\right\}$$
(ii) Total bending moment or rotation of the cross-section vanishes at the end of the beam:
  $$M+P^{H}+Q^{H}-M_{,1}^{H}=0\;\;\;\;\;\;\;\mathrm{or}\;\;\;\;\;\;\;\phi =0\;\;\;\;\mathrm{at}\;\;\;x_{1}\in \left\{0,\;L\right\}\;\;\;$$
(iii) Higher-order shear force or the deflection slope vanishes at the end of the beam:
  $$Q^{H}+N^{H}w_{,1}=0\;\;\;\;\;\;\;\mathrm{or}\;\;\;\;\;\;\;w_{,1}=0\;\;\;\;\;\mathrm{at}\;\;\;x_{1}\in \left\{0,\;L\right\}$$
(iv) Higher-order bending moment or gradient of rotation vanishes at the end of the beam:
  (29) $$M^{H}=0\;\;\;\;\;\;\mathrm{or}\;\;\;\;\;\;\;\phi_{,1}=0\;\;\;\;\;\mathrm{at}\;\;\;x_{1}\in \left\{0,\;L\right\}.$$

Substituting Equations (19), (20), (22) and (23) into (25), we obtain:

(30) $$M=\int_{A}\sigma_{11}x_{3}dA=-\left(c_{11}+\frac{e_{31}e_{31}}{a_{3}}\right)I\phi_{,1}=-S\phi_{,1},\;Q=\int_{A}\sigma_{13}dA=c_{44}A(w_{,1}-\phi ),\;N=\int_{A}\sigma_{11}dA=\frac{SA}{2I}{\left(w_{,1}\right)}^{2}-G\phi_{,1}+\Pi w_{,11},\;M^{H}=\int_{A}\tau_{111}x_{3}dA=-l^{2}c_{11}I\phi_{,11}=-Y\phi_{,11},\;Q^{H}=\int_{A}\tau_{131}dA=-\Psi \phi_{,1}+\Omega w_{,11}+\frac{\Pi }{2}{\left(w_{,1}^{}\right)}^{2}\;N^{H}=\int_{A}\tau_{111}dA=l^{2}c_{11}Aw_{,1}w_{,11}=\frac{AY}{I}w_{,1}w_{,11},\;P^{H}=\int_{A}\tau_{113}dA=-Z\phi_{,1}+Uw_{,11}+\frac{T}{2}{\left(w_{,1}^{}\right)}^{2}$$

Next, notations are used for the second moment of the cross-section area:

$I=\int_{A}{\left(x_{3}^{}\right)}^{2}dA$ and $S=\left(c_{11}+\frac{e_{31}e_{31}}{a_{3}}\right)Ip$,

$$Y=l^{2}c_{11}I,\;Z=\left(l^{2}c_{11}+\frac{f_{1}f_{1}}{a_{3}}\right)A+U,\;U=\frac{f_{1}f_{2}}{a_{3}}A,\;T=\frac{Ae_{31}}{a_{3}}f_{1},\;\Psi =\Omega +U,\;\Omega =\left(l^{2}c_{44}+\frac{f_{2}f_{2}}{a_{3}}\right)A,\;\Pi =\frac{Ae_{31}}{a_{3}}f_{2},\;G=T+\Pi$$

Substituting (30) into (28), a nonlinear system of ordinary differential equations is obtained:

(31) $$Y\phi_{,1111}-\Lambda \phi_{,11}+\Psi w_{,111}+c_{44}A(\phi -w_{,1})+(\Pi +T)w_{,1}w_{,11}=0,$$

(32) $$\begin{array}{l} \Psi \phi_{,111}+c_{44}A(w_{,11}-\phi_{,1})-\Omega w_{,1111}-G\left(\phi_{,11}w_{,1}+\phi_{,1}w_{,11}\right)\\ +\left(3\frac{SA}{2I}w_{,11}-\frac{AY}{I}w_{,1111}\right){\left(w_{,1}\right)}^{2}-\frac{AY}{I}\left({\left(w_{,11}\right)}^{3}+4w_{,1}w_{,11}w_{,111}\right)=-q \end{array}$$

where Λ=Z+S+Ψ.

For the beam subjected to the uniform transverse load and clamped at the end $x_{1}=0$, while for the free end at $x_{1}=L$, the following boundary conditions are prescribed:

(33) $$\begin{array}{l} {w|}_{x_{1}=0}=0,\;{w_{,1}|}_{x_{1}=0}=0,\;{\phi |}_{x_{1}=0}=0,\;{\phi_{,1}|}_{x_{1}=0}=0,\\ {\left[Q+Nw_{,1}+N^{H}w_{,11}-{\left(Q^{H}+N^{H}w_{,1}\right)}_{,1}\right]|}_{x_{1}=L}=0,\;{\left(Q^{H}+N^{H}w_{,1}\right)|}_{x_{1}=L}=0,\\ {\left(M+P^{H}+Q^{H}-M_{,1}^{H}\right)|}_{x_{1}=L}=0,\;{M^{H}|}_{x_{1}=L}=0. \end{array}$$

In order to solve the above nonlinear boundary value problem, we employ the weak formulation:

(34) $$\begin{array}{l} -\int_{0}^{L}\left[{\left(M+P^{H}+Q^{H}-M_{,1}^{H}\right)}_{,1}-Q\right]\delta \phi dx_{1}\\ +\int_{0}^{L}\left\{{\left[Q+Nw_{,1}+N^{H}w_{,11}-{\left(Q^{H}+N^{H}w_{,1}\right)}_{,1}\right]}_{,1}+q\right\}\delta wdx_{1}=0 \end{array}$$

with the finite element approximation for the field vector as $\left\{u\right\}={\left[\begin{array}{cc} w & \phi \end{array}\right]}^{T}$. After discretization, Equation (34) leads to the system of nonlinear algebraic equations, which can be written for the vector of nodal values $\left\{U\right\}={\left[\begin{array}{cc} \left\{W\right\} & \left\{\Phi \right\} \end{array}\right]}^{T}$ as:

(35) [K({U})]{U}={F}

Using the direct iteration technique (Picard iteration method), the solution at the *k*-th iteration is determined from the linearized equation:

(36) $$\left[K\left({\left\{U\right\}}^{\left(k-1\right)}\right)\right]{\left\{U\right\}}^{\left(k\right)}=\left\{F\right\},$$

where the coefficient matrix is evaluated using the known solution from the (*k* − 1)-st iteration. The initial guess vector ${\left\{U\right\}}^{\left(0\right)}$ is taken as the solution of equation $\left[\tilde{K}\right]{\left\{U\right\}}^{\left(0\right)}=\left\{F\right\}$, where $\left[\tilde{K}\right]$ is the coefficient matrix obtained from K({U}) by neglecting the nonlinear terms. The iterative procedure stopped when the difference between the two consecutive iterations, measured by the Euclidean norm, is less than the tolerance ε:

(37) $${\left(\frac{\left({\left\{U\right\}}^{\left(k\right)}-{\left\{U\right\}}^{\left(k-1\right)}\right){\left({\left\{U\right\}}^{\left(k\right)}-{\left\{U\right\}}^{\left(k-1\right)}\right)}^{T}}{{\left\{U\right\}}^{\left(k\right)}{\left({\left\{U\right\}}^{\left(k\right)}\right)}^{T}}\right)}^{1/2}\leq \varepsilon$$

## 4. Numerical Results

The piezoelectric material PZT-5H is considered in numerical examples with the following constants [35]:

(38) $$\begin{array}{l} c_{11}=12.6\times 10^{10}\;\mathrm{Pa},c_{13}=5.3\times 10^{10}\;\mathrm{Pa},c_{33}=11.7\times 10^{10}\;\mathrm{Pa},\\ c_{44}=3.53\times 10^{10}\;\mathrm{Pa},e_{31}=-6.5_{}\;{\mathrm{Cm}}^{-2},e_{33}=23.3_{}\;{\mathrm{Cm}}^{-2},\\ e_{15}=17.0_{}\;{\mathrm{Cm}}^{-2},a_{1}=15.1\times 10^{-9}\;C^{2}{/N/m}^{2},\;\\ a_{3}=13.0\times 10^{-9}\;C^{2}{/N/m}^{2},\;f_{1}=f_{2}=1\times 10^{-7}\;C/m \end{array}$$

and three various values are considered for the flexoelectric coefficients $f_{1}=f_{2}\in \left\{5,\;\;1,\;\;0.5\right\}\times 10^{-7}\;C/m$.

The length of the beam is *L =* 500 nm, the width is *B = H,* and the thickness is *H =* 20 nm. The microstructural length-scale parameter is chosen as $l=1\times 10^{-8}\;m$.

The weak form of the linearized differential Equation (34) has been implemented into the commercial software Comsol. The cubic Hermitian shape function has been used as interpolation functions and equidistantly distributed 1D finite elements.

The computational procedure for the 1D Euler–Bernoulli model is first verified for a linear model with the vanishing von Kármán strains. The convergence of the numerical results for deflection with respect to the increasing number of discretization elements is achieved for *N* > 50 finite elements, as shown in Table 1. In the following sections, we will use the finite elements with a length of 10 nm, when the convergence is guaranteed.

Moreover, the variation of the beam deflection along $x_{1}$ for various flexoelectric coefficients is obtained with the 2D analysis by the mixed FEM program [36], where 2000 rectangular elements are used. An opposite verification of the new computer code for the 2D problems by the Euler–Bernoulli theory was applied in [36] for a body force load, where the analytical solution is available.

**Figure 2:**
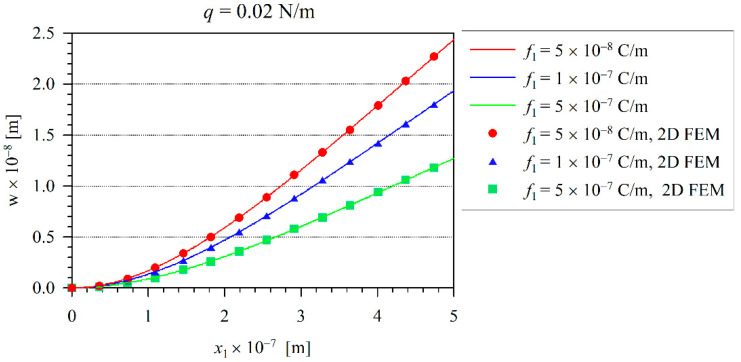
The variation of the deflection of the beam for various flexoelectric coefficients.

**Figure 3:**
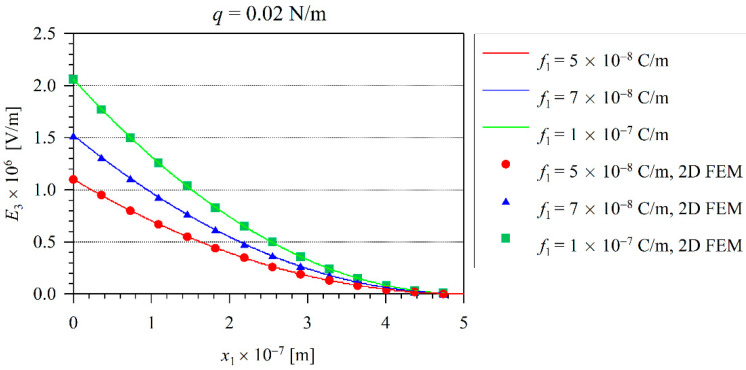
The distribution of the electric field $E_{3}$ along $x_{1}$ for a linear case.

The solid line in Figure 2 corresponds to the 1D Euler–Bernoulli model and the symbols are valid for the 2D FEM analysis. Here, one can observe the excellent agreement between the present numerical and analytical results. The induced electric field represented by the electric intensity $E_{3}$ on the neutral beam axis is presented in Figure 3. In addition, one can observe that the induced electric intensity increases with the increasing flexoelectric coefficients. It is in agreement with the results recently presented by the authors in [36]. The largest values of the electric intensity are at the clamped end, where the bending moment and the strain-gradients reach their maximum values. The vanishing electric potential is prescribed at the free end of the cantilever beam.

**Figure 4:**
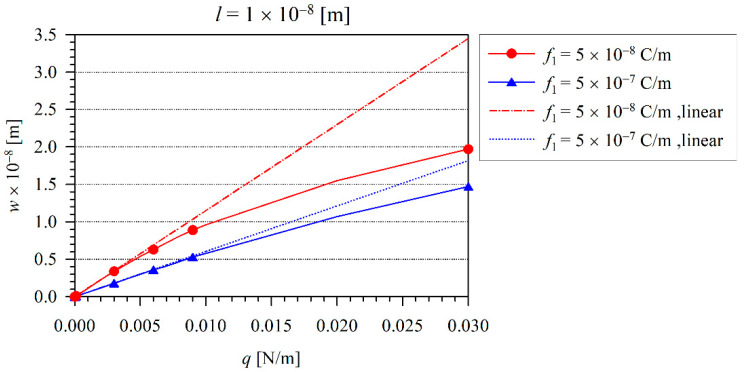
The variation of maximal beam deflection *w* with the load *q* for various flexoelectric coefficients and at the microstructural length-scale parameter $l=1\times 10^{-8}\;m$.

**Figure 5:**
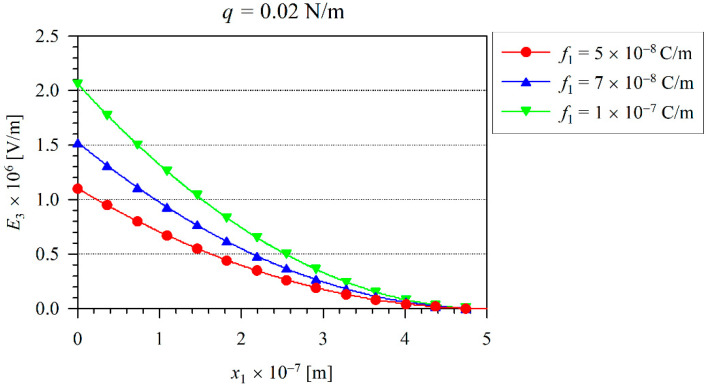
The variation of the electric field *E*_3_ along *x*_1_ for a nonlinear case.

Next, the effect of nonlinearity can be investigated. The iteration procedure is applied to consider the finite values of the von Kármán strains. For the iteration procedure, we have chosen the tolerance ε=0.005, which has been achieved after five iterations. The variation of the beam maximal deflection *w* with the load *q* for various flexoelectric coefficients and at the microstructural length-scale parameter $l=1\times 10^{-8}\;m$ is shown in Figure 4. The dashed line represents the deflection corresponding to the linear model. Here, one can observe that the decrease of nonlinear deflections in comparison with the linear ones is enhanced with the increasing load. It is in agreement with the observations in the classical theory of elasticity [37,38,39]. Similar to the linear elastic case, the increasing flexoelectricity reduces the beam deflection in the nonlinear case, as well. The variation of the electric intensity $E_{3}$ on the neutral beam axis is presented in Figure 5, when the load intensity is q=0.02 N/m. In Figure 4, one can see that at this load intensity, the nonlinearity is significantly large. From a comparison with Figure 5 (nonlinear case) and Figure 3 (linear case), one can see a significantly lower quantity of the induced electric intensity in the nonlinear case.

.

**Figure 6:**
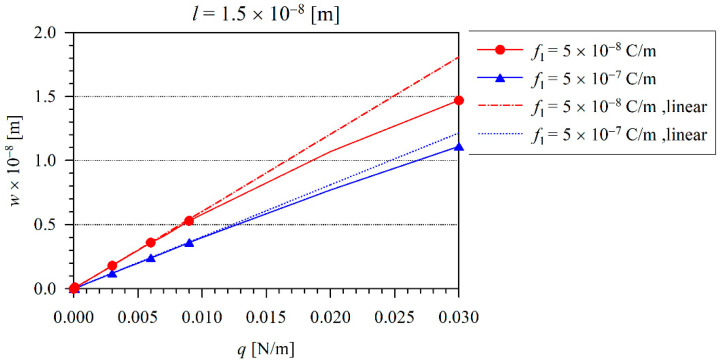
The variation of maximal beam deflection *w* with the load *q* for various flexoelectric coefficients and at the microstructural length-scale material parameter $l=1.5\times 10^{-8}\;m$.

Next, the influence of the microstructural length-scale parameter l on the beam deflection is investigated. If the value of this parameter grows by 50% with respect to the previous case in Figure 4, the beam deflection is further reduced as shown in Figure 4 and Figure 6. Evidently, the effect of nonlinearity is weaker if the intrinsic material parameter is larger.

**Figure 7:**
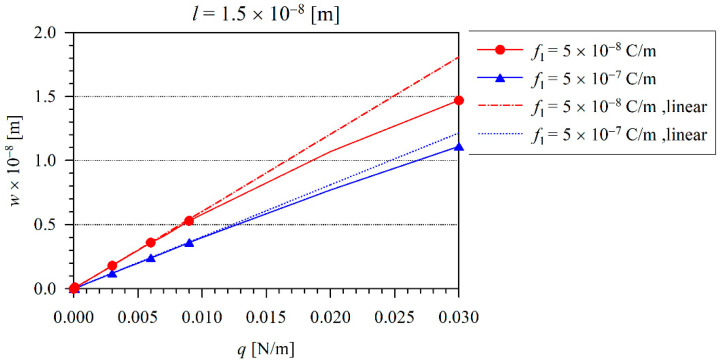
The variation of maximal deflection *w* with the intrinsic parameter *l* for two different loading levels and at the flexoelectric coefficient $f_{1}=5\times 10^{-8}\;C/m$.

The variation of maximal deflection *w* with the intrinsic parameter *l* for two different loading levels and at the flexoelectric coefficient $f_{1}=5\times 10^{-8}C/m$ is shown in Figure 7. Both of the curves for the two different load intensities are nearly parallel.

**Figure 8:**
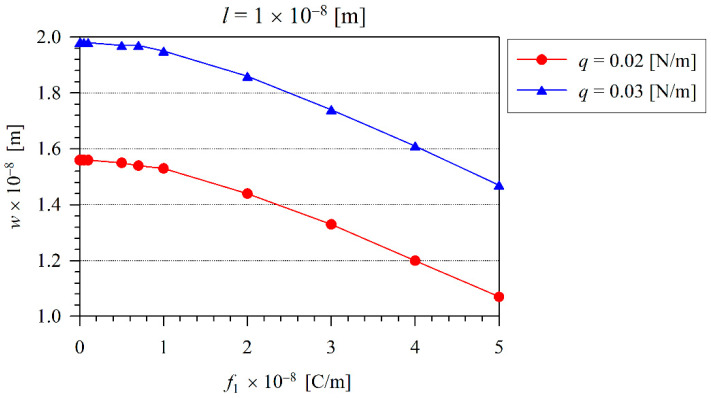
The variation of maximal deflection *w* with the flexoelectric coefficient for two different loading levels and at the microstructural length-scale parameter $l=1\times 10^{-8}\;m$.

**Figure 9:**
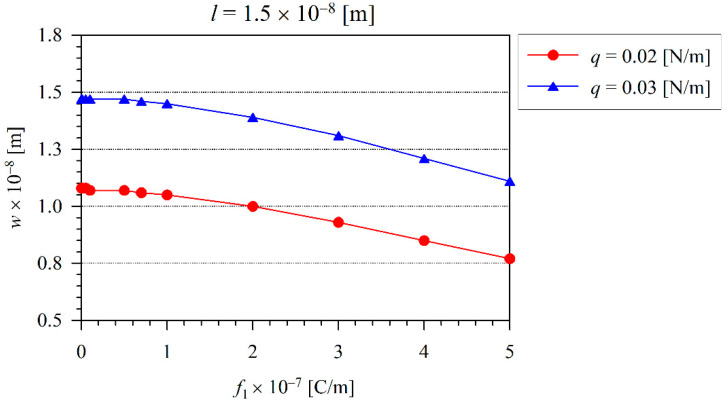
The variation of maximal deflection *w* with the flexoelectric coefficient for two different loading levels and at the microstructural length-scale parameter $l=1.5\times 10^{-8}\;m$.

The influence of the flexoelectric coefficient on the maximal beam deflection is clearly visible in Figure 8 and Figure 9 for two different values of the microstructural length-scale parameter. Here, one can observe that the deflection response is lower for the beam with the larger value of the microstructural length-scale parameter. The beam deflection decreases with the increasing value of the flexoelectric coefficient.

## 5. Conclusions

In nano-sized structures, higher-order derivatives of strains require consideration due to the large gradients of deformations. The mechanical and electrical response of the cantilever beam on mechanical loading is investigated within the higher-grade electroelasticity, including the direct flexoelectricity and von Kármán nonlinear strain deformations. In addition, the bending of the beam is studied within the Timoshenko beam theory. The principle of virtual work is applied to derive a nonlinear system of ordinary differential equations (ODEs) for the coupled fields of the electric potential, beam deflection, and rotation. The FEM scheme is employed for discretization and approximation of spatial field variations with the resulting system of nonlinear algebraic equations, as solved by the iteration procedure.

From the numerical results obtained for the clamped beam, one can observe a reduction of the mechanical as well as electrical response of the beam within the nonlinear model, as compared with the linear one. Furthermore, the response decreases with the increasing level of the mechanical load. The flexoelectric effect consumes a portion of the energy of the external forces and effectively hinders the bending of the beam under a pure mechanical load. The intensity of the induced electric field rapidly grows with the increasing value of the flexoelectric coefficient.

## Figures and Tables

**Table 1:** Convergence of numerical results for the midpoint deflection with respect to the number of discretization elements.

Number of Elements	w (x_1_ = 2.5 × 10^−7^)
10 elements	6.353723 × 10^−9^
20 elements	6.750830 × 10^−9^
50 elements	6.750842 × 10^−9^
100 elements	6.750842 × 10^−9^

## Data Availability

Not applicable.
